# Neuroblastoma: an ongoing cold front for cancer immunotherapy

**DOI:** 10.1136/jitc-2023-007798

**Published:** 2023-11-22

**Authors:** Paul T Kennedy, Demetra Zannoupa, Meong Hi Son, Lekh N Dahal, John F Woolley

**Affiliations:** 1 Department of Pharmacology & Therapeutics, University of Liverpool, Liverpool, UK; 2 Department of Pediatrics, Samsung Medical Center, Gangnam-gu, Seoul, Korea (the Republic of)

**Keywords:** Neuroblastoma, Immunotherapy, Tumor Microenvironment

## Abstract

Neuroblastoma is the most frequent extracranial childhood tumour but effective treatment with current immunotherapies is challenging due to its immunosuppressive microenvironment. Efforts to date have focused on using immunotherapy to increase tumour immunogenicity and enhance anticancer immune responses, including anti-GD2 antibodies; immune checkpoint inhibitors; drugs which enhance macrophage and natural killer T (NKT) cell function; modulation of the cyclic GMP-AMP synthase-stimulator of interferon genes pathway; and engineering neuroblastoma-targeting chimeric-antigen receptor-T cells. Some of these strategies have strong preclinical foundation and are being tested clinically, although none have demonstrated notable success in treating paediatric neuroblastoma to date. Recently, approaches to overcome heterogeneity of neuroblastoma tumours and treatment resistance are being explored. These include rational combination strategies with the aim of achieving synergy, such as dual targeting of GD2 and tumour-associated macrophages or natural killer cells; GD2 and the B7-H3 immune checkpoint; GD2 and enhancer of zeste-2 methyltransferase inhibitors. Such combination strategies provide opportunities to overcome primary resistance to and maximize the benefits of immunotherapy in neuroblastoma.

## The immune landscape of neuroblastoma

Neuroblastomas are the most common extracranial solid tumour in children, with 90% of cases occurring in children less than 10 years of age.^1^ Deriving from neural-crest progenitor cells in the developing sympathetic nervous system, neuroblastomas are often located in the sympathetic ganglia and adrenal glands.^2^ A wide range of management strategies are employed depending on risk stratification, with some young infants receiving minimal intervention and experiencing spontaneous tumour regression, and other cases with high-risk pathology requiring intensive multimodal therapy, including radiotherapy, chemotherapy and surgery.^2^ Advances in treating neuroblastoma have improved the 5-year survival probability for high-risk disease from 29% for those diagnosed between 1990 and 1994, to 50% for patients diagnosed between 2005 and 2010^3^ Despite this, conventional therapies can induce long-term toxicities and often provide limited benefit, thus driving efforts to harness the immune system to control neuroblastoma.

High-risk neuroblastomas are classified as immunologically “cold” due to limited T-cell infiltration, major histocompatibility complex class I (MHC-I) downregulation and low mutational burden leading to an immunosuppressive tumour microenvironment (TME).^4^ The immune contexture of neuroblastomas comprises tumour associated macrophages (TAMs), natural killer (NK) cells, lymphocytes, dendritic cells (DCs), Schwann cells, cancer-associated fibroblasts (CAFs), vascular endothelial cells and mesenchymal cells.^5^ Regulatory T cells (T_reg_) exert highly immunosuppressive effects through functions including secretion of inhibitory cytokines, such as interleukin (IL)-10, which attenuate cytotoxic T-cell responses and inhibit antigen presenting cell maturation.^4 6^ However, in neuroblastoma the role of Tregs in the TME is unclear; increased Treg infiltration was shown to be associated with improved patient survival.^4^ CAFs represent the major non-tumour stromal cell type and regulate the TME and extracellular matrix (ECM).^6^ Cellular interactions between CAFs and TAMs induce mutual activation and signaling events which promote neuroblastoma progression.^5 6^ The presence of TAMs in the TME is associated with poor patient prognosis as secretion of a cocktail of inflammatory cytokines, such as IL-6 and vascular endothelial growth factors (VEGF), promotes angiogenesis and neuroblastoma proliferation, whilst having an immunosuppressive role by inhibiting the function of DCs, NK cells and T cells.^5 6^ Furthermore, the stiffness and rigidity of the ECM may exert physical forces on migrating tumour cells and induce chromosomal instability.^7^ Because of the poorly immunogenic TME, patients are often resistant to immune checkpoint blockade (ICB) therapy.

## Challenges to effective neuroblastoma immunotherapy

This review will address the shortcomings and advances in the use of immunotherapy for the treatment of neuroblastoma. The clinical characteristics and risk classifications for neuroblastoma have been extensively discussed elsewhere.^8–11^ The notoriously immunosuppressive neuroblastoma TME is resistant to conventional ICB monotherapy and represents the principal challenge to effective immunotherapy. Due to the poorly immunogenic features of neuroblastomas, alternative strategies involving adjuvant treatments are being investigated to induce sensitivity to ICB.^12^ Additionally, although anti-GD2 therapies can produce clear and durable responses in high-risk neuroblastoma, current treatment options carry a significant toxicity burden. Due to this, there is an urgent need to develop strategies which both extend survival and improve quality of life for patients with high-risk neuroblastoma.

**Figure 1:**
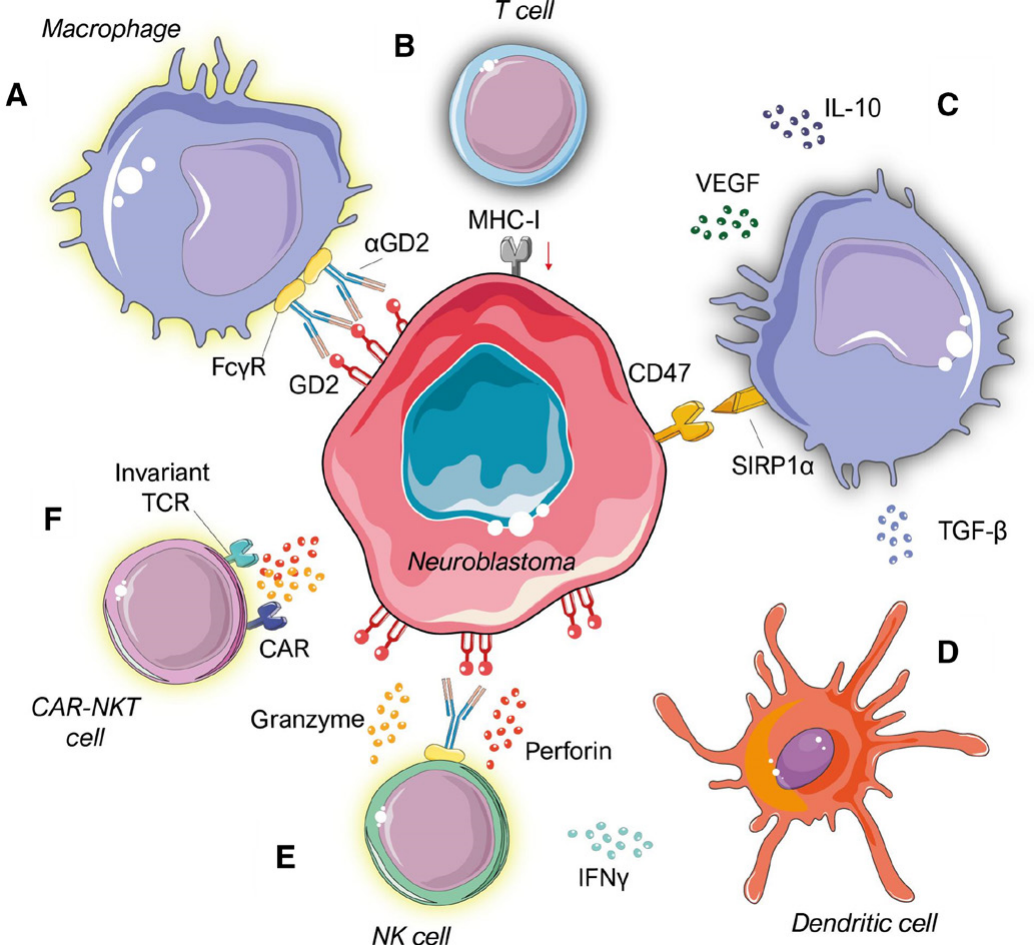
Targets for immunotherapy within the neuroblastoma tumour microenvironment. (A) GD2 expression represents an attractive therapeutic target due to ubiquitous and abundant expression in neuroblastoma regardless of disease stage, in addition to limited expression in other cell types. (B) Targeting neuroblastoma specific GD2 expression with monoclonal antibodies promotes tumour cell phagocytosis by macrophages *via* antibody dependent cellular cytotoxicity. The neuroblastoma tumour microenvironment often lacks significant infiltration and activation of T cells and is instead dominated by tumour-promoting stromal cells such as tumour-associated macrophages. Amplification of the *MYCN* oncogene is often associated with reduced major histocompatibility complex class I (MHC-I) expression, which reduces presentation of tumour-associated antigens and compromises T-cell recognition and elimination of neuroblastomas. (C) Neuroblastoma overexpression of “don’t eat me” signal CD47 inhibits effective phagocytosis by macrophages through signal regulatory protein alpha (SIRP1α) interaction. TAMs also secrete cocktails of chemokines and cytokines, such as VEGF and the potently immunosuppressive IL-10, which promote immune evasion and tumour growth. (D) Dendritic cells secrete pro-inflammatory cytokines which activate NK cells in the neuroblastoma TME. (E) NK cells also facilitate antibody-dependent cellular cytotoxicity and can target neuroblastoma via MHC-I-independent cytotoxic mechanisms. Secretion of cytolytic granules granzyme-B and perforin induces tumour cell lysis.(F) Adoptive transfer of molecularly engineered CAR-NKT cells enhances NK-cell mediated targeting and clearance of neuroblastoma cells. CAR, chimeric-antigen receptor; IFN, interferon; IL, interleukin; NK, natural killer; NKT, natural killer T cell; TCR, T cell receptor; TGF, transforming growth factor; VEGF, vascular endothelial growth factors.

Amplification of the *MYCN* oncogene, which is frequently seen in patients with high-risk neuroblastoma, is a major driver of poor prognosis and is associated with decreased tumour immunogenicity.^4 8 13^ Moreover, *MYCN* amplification is responsible for the suppression of interferon activity as well as the expression of chemokines, and its overexpression enhances tumour cell resistance to immune-mediated cytotoxicity via multiple mechanisms, including MHC-I downregulation and inhibition of NK cell activation.^4 14^ Several ongoing clinical trials involving immunotherapy for neuroblastoma (table 1) are assessing the efficacy of novel combinations of immunomodulatory and conventional anticancer therapeutics, however many remain early-phase with no published results. Here, we will discuss the success and shortcomings of treatments which have progressed through clinical testing, in addition to the rationale and potential of novel immunotherapy strategies for neuroblastoma (figure 1).

**Table 1:** Clinical trials for neuroblastoma immunotherapies

Trial	Phase	Drugs	Target	Reference
NCT03363373	II	GM-CSF+naxitamab	GD2	33
NCT01757626	I/II	GM-CSF+naxitamab	GD2	26
NCT00072358	II	3F8	GD2	105 106
NCT00492167	I	Beta-glucan+3F8	GD2	107
NCT02914405	I	Nivolumab+dinutuximab beta	GD2+PD-1	
NCT01704716	III	Dinutuximab beta+IL-2	GD2	35
NCT04751383	I	Magrolimab+dinutuximab	GD2+CD47	68 ^†^
NCT03189706	Early I	Naxitamab+GM-CSF	GD2	
NCT04501757	*	Naxitamab+GM-CSF	GD2	
NCT03033303	II	Naxitamab+GM-CSF+isotretinoin	GD2	
NCT02650648	I	NK cells+naxitamab	GD2+NK cells	89 ^†^
NCT01662804	I	IL2+naxitamab	GD2	
NCT01419834	I	Naxitamab	GD2	
NCT03332667	I	Dinutuximab+131I-MIBG	GD2	108
NCT01711554	I	Lenalidomide+dinutuximab	GD2+NK cells	109
NCT02573896	I	Lenalidomide+dinutuximab	GD2+NK cells	
NCT03786783	II	Dinutuximab+sargramostim	GD2	
NCT01857934	II	hu14.18K322A+GM-CSF/G-CSF+IL2+NK cell infusion	GD2+NK cells	38 79
NCT04385277	II	Dinutuximab+sargramostim+isotretinoin	GD2	
NCT00026312	III	Isotretinoin+dinutuximab+aldesleukin	GD2	18
NCT01445379	I	Ipilimumab	CTLA-4	51
NCT02813135	I/II	Nivolumab	PD-1	45
NCT02304458	I/II	Nivolumab+ipilimumab	PD-1+CTLA-4	44
NCT02914405	I	Nivolumab+dinutuximab beta	PD-1+GD2	
NCT04412408	Early I	Sintilimab	PD-1	
NCT04730349	I/II	Nivolumab+bempegaldesleukin	PD-1	
NCT05302921	II	Nivolumab+ipilimumab	PD-1+CTLA-4	
NCT02541604	I/II	Atezolizumab	PD-1	47
NCT00877110	I	3F8+allogeneic NK cells	NK cells+GD2	89
NCT03209869	I	Expanded NK cells+Hu14.18-IL2	NK cells+GD2	
NCT03294954	I	Anti-GD2 CAR-NK cells	GD2+NK cells	88
NCT03242603	I/II	Anti-GD2+NK cells	GD2+NK cells	110

*Approved for marketing.

†Study referenced done prior to the clinical trial.

CAR, chimeric-antigen receptor; CSF, colony stimulating factor-1 ; CTLA-4, cytotoxic T-lymphocyte associated protein-4; GM-CSF, granulocyte-macrophage colony-stimulating factor ; IL, interleukin; NK, natural killer; PD-1, programmed cell death protein-1.

## Targeting GD2 in neuroblastoma treatment

GD2 is a disialoganglioside highly expressed in melanomas and neuroblastomas, implicated in tumour progression by modulating cancer cell proliferation, migration and tumour invasiveness.^15–17^ Since its expression is highly restricted to few normal cell types such as neurons, skin melanocytes and peripheral sensory fibers, GD2 represents a particularly attractive tumour-associated antigen for monoclonal-antibody-based targeting and killing of neuroblastomas via antibody-dependent cellular cytotoxicity (ADCC).^18^ Furthermore, GD2 is expressed ubiquitously at high density in primary neuroblastomas regardless of disease stage, increasing its utility as an antigen for targeted immunotherapy.^19^

### 3F8

The murine IgG3 antibody 3F8 is the first anti-GD2 antibody developed and studied for effectiveness in treating neuroblastoma. An early phase I study of anti-GD2 monotherapy showed anti-tumour responses, ranging from complete clinical remission to mixed tumour regressions, in 7 of the 17 patients with neuroblastoma and melanoma treated. Typical acute side effects of severe pain and urticaria were prevalent throughout this study, thought to be the result of 3F8 interaction with pain sensory fibers, but long-term effects were not observed.^20^ Despite demonstrating effectiveness against minimal residual disease, studies of 3F8 have failed to produce notable efficacy against bulky disease.^21 22^ Due to observations that human anti-mouse antibody (HAMA) responses following treatment correlated with reduced persistence of anti-GD2 antibodies in circulation,^23 24^ a humanized 3F8 was developed (hu3F8) to overcome potential reductions in ADCC and complement-dependent cytotoxicity (CDC).^25^

### Naxitamab

Naxitamab (Hu3F8) is a humanized IgG1 antibody being tested clinically as both a monotherapy and in combinations with immunogenic cytokines (table 1). A phase I dose-escalation study (NCT01757626) of naxitamab, in combination with granulocyte-macrophage colony-stimulating factor (GM-CSF) to stimulate monocyte production and maturation, demonstrated modest and manageable toxicity with no maximum tolerated dose, in addition to significant anti-tumour responses and durable progression-free survival (PFS) achieved even in patients with refractory disease.^26^ Following striking results from a phase II multicenter trial (NCT03363373) showing naxitamab treatment induced a 61% complete response rate in high-risk patients with refractory or relapsed bone disease, naxitamab with GM-CSF received Food and Drug Administration approval for this indication.^27^ Interestingly, many trial participants were managed as outpatients due to easily manageable toxicities, indicating better tolerance to humanized anti-GD2 compared with the murine m3F8. Despite this, naxitamab treatment typically requires supervision and further intervention to manage severe pain and hypotension.

### Dinutuximab

Several anti-GD2 antibodies have been generated and characterized over the past decade, with dinutuximab (Ch14.18/SP2/0) being the first to receive marketing approval in 2015 for the treatment of high-risk neuroblastoma.^28^ It is a chimeric IgG1 anti-GD2 monoclonal antibody derived from SP2/0 hybridoma cells and received approval after a phase III clinical trial (NCT00026312) demonstrated that patients receiving a combination of GM-CSF, IL-2, isotretinoin and dinutuximab exhibited significantly better PFS and overall survival (OS) compared with those treated with standard chemotherapy.^29^ However, significant toxicities were reported throughout this study, with a high frequency of participants exhibiting high-grade pain, capillary leak syndrome and serious infusion reactions.^18^ Toxicity is often so severe that side effects require careful management with other pharmaceuticals such as analgesics. Long-term follow-up revealed late-relapse in a subset of patients, although benefit to survival outcome across the cohort remained significant.^29^

### Dinutuximab beta

Dinutuximab beta (Ch14.18/CHO) is a dinutuximab analog produced by the International Society of Paediatric Oncology European Neuroblastoma Group (SIOPEN) using Chinese hamster ovary (CHO) cells to optimize protein glycosylation patterns and reduce rapid drug clearance by endogenous antibodies which recognize non-human sialic acid epitopes in antibodies derived from SP2/0 cells.^30^ The re-cloned Ch14.18/CHO exhibited similar pharmacokinetics in both humans and mice, with a small-scale phase I trial from SIOPEN showing similar toxicity profiles and evidence of clinical neuroblastoma responses.^30^ Dinutuximab beta is approved as a second-line treatment for patients with high-risk neuroblastoma over 12 months of age who initially responded to induction chemotherapy, or in combination with IL-2 for those with refractory disease.^31^ Incorporation of IL-2 is supported by preclinical and clinical studies showing an enrichment of activated NK cells enhances anti-GD2-mediated ADCC.^32 33^ Interestingly, a phase III multicenter trial (NCT01704716) found that inclusion of subcutaneous IL-2 with dinutuximab beta did not improve the survival of patients with prior responses to standard induction therapy, but significantly increased the incidence of toxicities such as hypersensitivity and neurotoxicity.^34^ A follow-up study showed that decreasing the dose of IL-2 significantly reduced associated toxicities.^35^ Together they support the reservation of IL-2 as an adjuvant treatment for particularly hard-to-treat tumours. Although fine-tuning of immunomodulatory treatments to maximize anti-tumour responses while avoiding immune-related adverse events is highly challenging, optimization of treatment schedules may yet improve clinical responses to dinutuximab in neuroblastoma.

### hu14.18K322A

Other anti-GD2 antibodies in development have followed a similar approach of humanization to counteract dose-limiting toxicities and HAMA responses. Indeed, phase I clinical testing of humanized 14.18 (IgG1 isotype, hu14.18K322A) demonstrated reduced pain and other toxicities associated with ch14.18 treatment, while still inducing an objective response in 18% of patients with refractory neuroblastoma.^36^ Efforts to augment anti-neuroblastoma responses progressed from subcutaneous administration of IL-2 to the design of a hu14.18-IL-2 fusion protein. A phase II trial of hu14.18-IL-2 monotherapy in patients with refractory neuroblastoma demonstrated complete responses (21.7%) in patients with minimal residual disease, although patients with bulky disease did not respond.^37^ The study suggested that this antibody when used along with induction chemotherapy improves early responses, reduces tumour volume and leads to an event-free survival of 3 years.^38^

Overall, multiple anti-GD2 antibodies have undergone molecular revision and refinement to reduce immune-related toxicities and severe neuropathic pain associated with CDC and HAMA responses. Despite the attractive tissue expression and restricted distribution of GD2 as a target, the ADCC-based mechanism of anti-GD2 induces often intolerable toxicity made worse by combination with activating cytokines such as IL-2. Alternative methods of enhancing anti-GD2 efficacy are required, since of the three clinically approved antibodies available, currently dinutuximab is the only one deemed effective for standard therapy.

## Immune checkpoint blockade for neuroblastoma treatment

Immune checkpoint proteins are expressed by a range of immune cell types and perform critical functions in maintaining tolerance and preventing autoimmunity.^39^ Although there are multiple receptor-ligand interactions that constitute immune checkpoints, programmed cell death protein-1 (PD-1)/programmed death-ligand 1 (PD-L1) and cytotoxic T-lymphocyte associated protein-4 (CTLA-4)/B7 represent the most widely studied mechanisms in this category of immunomodulatory processes. PD-1 signaling regulates numerous immunological processes, including T-cell activation, cytolytic activity, trafficking and migration.^40^ Similarly to PD-1, CTLA-4 is upregulated upon T-cell activation but exhibits a distinct checkpoint mechanism and attenuates T-cell function by competing for B7 ligands to reduce co-stimulatory signal strength.^41 42^ Although PD-1 and CTLA-4 perform critical functions in maintaining tissue homeostasis, they are often co-opted during tumourigenesis and represent a targetable hallmark of many cancer types.^43^ However, despite the ICB becoming a paradigm-shifting tool in effective cancer immunotherapy, little progress has been made in exploiting it for successful neuroblastoma treatment.

### Targeting PD-1

The fully human IgG4 antibody nivolumab was evaluated in two phase II trials designed to assess the toxicity and pharmacokinetics of anti-PD-1 monotherapy in patients with refractory neuroblastoma (NCT02304458, NCT02813135). The first study demonstrated that nivolumab is well-tolerated in children with similar pharmacokinetics to those seen in adults, however objective responses were not observed and benefit was limited to stable disease only.^44^ The second study showed that use of nivolumab monotherapy was ineffective,^45^ which is consistent with the results of preclinical studies of single-agent immune checkpoint inhibitors (ICIs).^46^ Another monoclonal antibody used clinically to target the PD-1 pathway in a range of adult cancers is the humanized IgG1 anti-PD-L1 antibody atezolizumab. A phase I/II paediatric study of atezolizumab in children with solid tumours such as neuroblastoma (NCT02541604) demonstrated safety but limited anti-tumour responses.^47^ Thus, PD-1/PD-L1 blockade in paediatric patients with neuroblastoma has proven ineffective in clinical trials to date, despite the significant clinical successes in adult cancers. This may be a result of trial design and patient recruitment since adults approved for treatment with nivolumab or atezolizumab undergo pathology assessment to determine tumour mutational burden (TMB) and PD-L1 positivity. A caveat of the trials discussed is that the patients with neuroblastoma recruited were known to exhibit tumours with low TMB and low PD-L1 expression, so the lack of sensitivity to ICB is unsurprising.^48^ Future trials with specific ICI-related eligibility criteria may reveal a subset of patients sensitive to PD-1/PD-L1 blockade; potentially those exhibiting mismatch repair deficiency and a sufficient antigen load to drive anti-tumour T-cell responses.

Preclinical studies have sought to overcome the challenge of low-sensitivity to anti-PD-1 in neuroblastoma by inducing target cell PD-L1 expression. One provocative study demonstrated that targeting GD2 in LA-N-1 neuroblastoma cells with ch14.18/CHO in co-culture with primary leukocytes induced strong PD-L1 expression in what is usually a PD-L1 negative model.^49^ Interestingly, LA-N-1 PD-L1 upregulation effectively inhibited ch14.18-mediated ADCC, an effect abrogated by nivolumab treatment. Indeed, the nivolumab-ch14.18 combination synergistically inhibited the growth of NXS2-HGW murine neuroblastoma tumours in vivo and enhanced survival relative to either agent alone.^49^ This strategy was tested clinically in an interesting case involving two heavily pretreated patients with refractory disease.^50^ Strikingly, one patient exhibited a complete response and regression of multiple positron emission tomography (PET)-positive lesions, whilst the other exhibited tumour shrinking and partial remission. Although this was a minor case study, the observations are notable for the responses achieved in patients previously unresponsive to dinutuximab and potentially indicate that anti-GD2-induced PD-L1 expression renders neuroblastoma susceptible to nivolumab treatment.^50^

### Targeting CTLA-4

Ipilimumab is a first-in-class human monoclonal antibody targeting the immune checkpoint CTLA-4. A phase I clinical trial (NCT01445379) assessing pharmacokinetics and immunogenicity in a mixed cohort off refractory solid tumours showed that it is tolerated in children, although immune-related adverse events (irAEs) consistent with those seen in adults were observed. Despite the lack of tumour regressions following single-agent ipilimumab, patients who exhibited irAEs had an increased OS relative to those who did not, suggesting that disruption of immunological tolerance with ipilimumab was beneficial in terms of survival outlook.^51^

### Targeting both PD-1 and CTLA-4

Poor clinical responses to single-agent ICB have motivated preclinical investigations into combination strategies. One such study showed that the murine syngeneic NB-SQ neuroblastoma model exhibits an immunosuppressive TME characterized by a high frequency of myeloid-derived suppressor cells and Tregs.^52^ Consistent with clinical studies described here, the treatment of NB-SQ tumours with single-agent anti-PD-1 or anti-CTLA-4 failed to control the growth of established tumours. Notably, mice treated with dual anti-PD-1/anti-CTLA-4 following a course of cyclophosphamide exhibited an enrichment of effector CD8+T cells and significantly increased survival compared with those treated with ICI monotherapy.^52^ An alternative preclinical study evaluated an induced neuroblastoma model with immunogenic features and high-level PD-L1 expression.^53^ The authors observed that the murine N2a cell line mimics features of human SK-N-SH and SH-SY5Y neuroblastoma cells and that each cell line exhibits interferon (IFN)-γ-inducible PD-L1 expression. Interestingly, subcloning of N2a produced an aggressive AgN2a model which lacked PD-L1 expression and sensitivity to IFN-γ, reflecting features of high-risk clinical neuroblastoma. Combining anti-PD-1 or anti-CTLA-4 with tumour cell vaccines for these neuroblastoma cell lines induced complete regression of established tumours in vivo. It should be noted that ipilimumab with nivolumab is approved for the treatment of unresectable melanoma, and given that one safety trial has established comparable safety profiles for ipilimumab in adults and children, this represents a potential avenue for future progress.

## Enhancing neuroblastoma cell phagocytosis by tumour-associated macrophages

**Figure 2:**
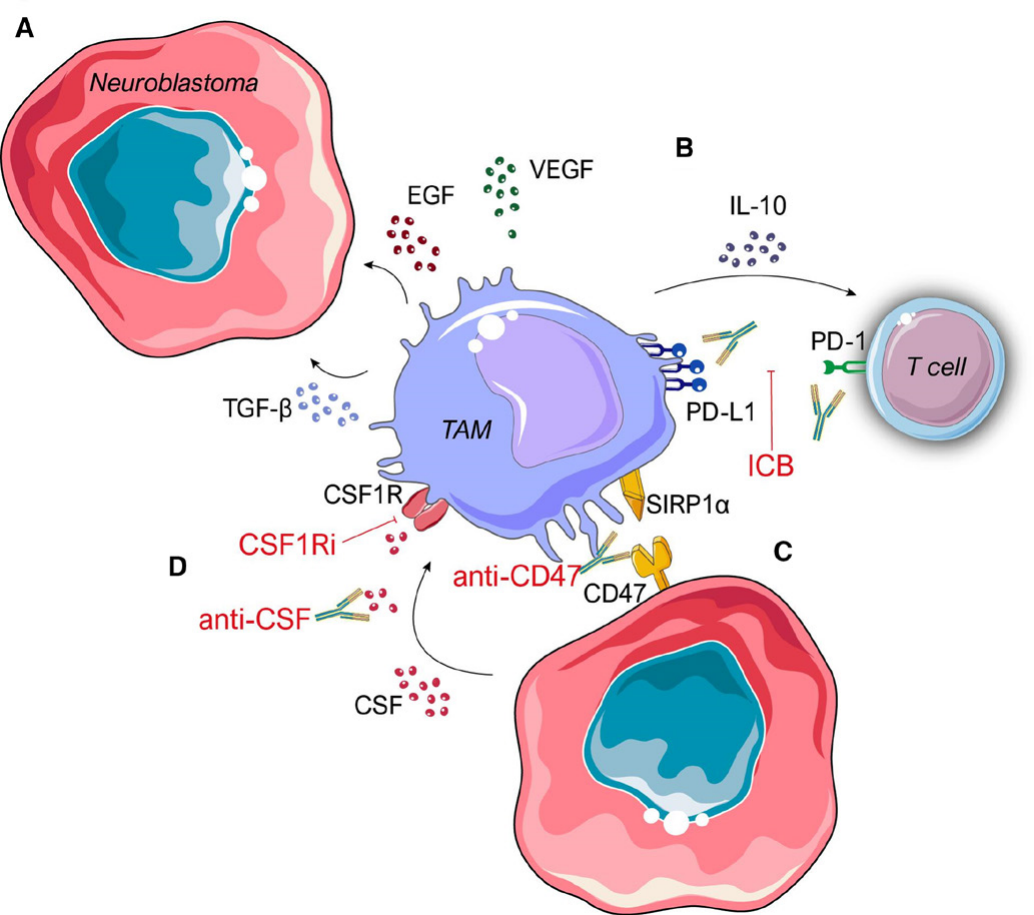
tumour associated macrophages as therapeutic targets in neuroblastoma. (A) tumour associated macrophages (TAMs) secrete stroma-regulating mediators such as matrix metalloproteinases, vascular endothelial growth factors (VEGF), endothelial growth factors (EGF) and chemokines (CCL2) which promote cancer cell growth and proliferation, angiogenesis, and tumour metastasis. (B) TAM-derived interleukins (IL-9 and IL-10), growth factors (TGF-β) and chemokines (CXCL9 and CXCL10) attenuate T-cell effector responses. T-cell effector responses may be promoted using monoclonal antibodies for immune checkpoint blockade (ICB), such as the anti-PD-1 antibody pembrolizumab. (C) CD47 expression masks neuroblastoma cells from surveillance and targeting by phagocytes. Anti-CD47 antibodies reduce tumour immune evasion and enhance cancer cell phagocytosis by macrophages. (D) Neuroblastoma cells secrete colony stimulating factor 1 (CSF-1) to promote TAM recruitment to the tumour microenvironment. Since macrophage infiltration is a poor prognostic indicator in neuroblastoma, anti-CSF antibodies may inhibit CSF-1R activation and cell trafficking. Immunotherapy strategies are indicated in red. PD-1, programmed cell death protein-1; PD-L1, programmed death-ligand 1; SIRP1α, signal regulatory protein alpha.

The importance of TAMs in TME of neuroblastoma and other cancers is well established.^54–56^ Macrophages can be simplistically classified into two groups; M1, which exert pro-inflammatory functions and alternatively activated M2, which secrete anti-inflammatory cytokines.^57^ TAMs are functionally similar to the M2 macrophages as they support tumourigenesis by secreting chemokines and ILs such as IL-10 (figure 2).^57^ Immune editing processes in neuroblastoma sculpt a TME dominated by immunosuppressive cell types, including TAMs, which inhibit effective anti-tumour T-cell responses.^58 59^ Surveillance of the TME by macrophages relies on the signal regulatory protein alpha (SIRPα)-CD47 axis to prevent aberrant phagocytosis of healthy host cells. However, CD47 upregulation is another mechanism by which tumour cells can subvert immune surveillance, by delivering “don’t eat me” signals to TAMs.^60^ Indeed, CD47 upregulation is often observed in neuroblastomas, rendering them resistant to phagocytosis.^61 62^

### Targeting CD47 to promote phagocytosis

In contrast to GD2, the transmembrane integrin-associated protein CD47 is ubiquitously expressed throughout a range of different tissue types. Being frequently overexpressed in tumours, CD47 enrichment is strongly associated with worse prognosis in both haematological and solid cancers.^63^ Inhibition of CD47 promotes macrophage-mediated phagocytosis of target tumour cells which also express secondary pro-phagocytic signals, such as calreticulin.^64^ Magrolimab (Hu5F9-G) is a first-in-class humanized IgG4 anti-CD47 which exhibits ICI activity in a wide range of preclinical solid tumour models.^63 65^ A first-in-human phase I trial of magrolimab (NCT02216409) revealed mild-to-moderate toxicity and objective tumour responses were reported in two patients, one with ovarian and another with fallopian tube tumours.^63^ A preclinical study demonstrated potent Hu5F9-G4 activity against multiple patient-derived xenograft models of paediatric brain tumours, observing substantial macrophage recruitment in treated tumours.^66^ An important finding of this study was that Hu5F9-G4 did not induce toxicity in neural progenitor cells, or promote phagocytosis of differentiated progeny, indicating differentiation between normal and cancer cells. Several drugs targeting the CD47-SIRPα axis are being tested against multiple tumour types; these include Hu5F9, TTI-621, ALX-148, AO-176 and OSE-172.^67^ Of these, only Hu5F9 is currently being studied for neuroblastoma treatment in combination with dinutuximab in a first-in-human/first-in-child phase I trial (NCT04751383). Although no clinical data has been released, recent assessment of this strategy in preclinical xenograft and murine syngeneic neuroblastoma models demonstrated potent synergy and tumour clearance.^68^ Notably, the authors identified the likely synergistic mechanism as GD2 ligation-dependent upregulation of the pro-phagocytic signal calreticulin, in addition to blocking GD2 interactions with the inhibitory immunoreceptor Siglec-7. Indeed, these encouraging findings are supported by other preclinical studies which demonstrated CD47 upregulation following anti-GD2 treatment functions as an adaptive immune checkpoint which limits effective ADCC in neuroblastoma models.^69^ Thus, dual targeting of GD2 and CD47 represents a promising strategy for clinical translation in neuroblastoma.

### Targeting the colony stimulating factor-1 signaling

Colony stimulating factor-1 (CSF-1)/CSF-1R interactions can also be targeted to modulate TAM migration and accumulation in tumour sites.^57^ CSF-1R monoclonal antibodies such as RG7155 starve CSF-1-dependent macrophages of a crucial growth factor and suppress the growth of multiple cancers.^60^ A preclinical study assessing the effect of the selective CSF-1R inhibitor BLZ945 against the transgenic spontaneous TH-*MYCN* model of high-risk neuroblastoma demonstrated significant growth-limiting effects after treating established tumours.^70^ Interestingly, intratumoural macrophage and monocyte densities were unaffected by BLZ945, but significant depletion of splenic macrophages was observed, potentially indicating myeloid sustenance by tumour-cell derived GM-CSF.^71^ Due to observations that intratumoural myeloid cells are a significant source of stromal PD-L1, and that CSF-1R inhibition increases T-cell PD-1 expression, the same study assessed the effect of ICB on BLZ945 activity. Treatment with ICIs alone had no effect on tumour growth, while combination with BLZ945 induced potent synergy and complete regression of small tumours and inhibited progressive growth of large tumours in a majority of mice.^70^ Since CSF-1R^+^ myeloid infiltration predicts poor neuroblastoma outlook and PD-L1 is strongly associated with CSF-1R expression in human neuroblastomas, this may represent a promising therapeutic avenue.^72^ However, preclinical evidence for CSF-1R inhibition reported to date is often conflicting and highly model and context-dependent. Some studies report dramatic remodeling of the myeloid compartment, but only moderate effects on tumour growth,^73^ while others report significant anti-tumour activity following intratumoural macrophage repolarization.^74^

Clinical trials aiming to validate the therapeutic benefit observed with preclinical CSF-1R-PD-1 dual inhibition using both small molecule (NCT02452424) and monoclonal antibody inhibitors (NCT02526017 and NCT02323191) are assessing responses in patients with neuroblastoma.

## Targeting natural killer cells

NK cells can target tumour cells for cytolytic killing and are known to produce pro-inflammatory cytokines and chemokines which attenuate tumour progression and metastasis.^75^ NK cells have a significant role in facilitating ADCC and are one of the main effector populations involved in immunotherapeutic mechanisms, thus are a major consideration when designing monoclonal antibody therapies for cancer.^75 76^ Specifically, preclinical studies have shown that the anti-neuroblastoma activity of the anti-GD2 antibody ch14.18 is dependent on NK cells, since NK cell depletion abrogated the efficacy observed.^77^ Efforts to boost NK cell anti-tumour activity include treatment with IL-2 or promoting NK cell-DC interactions.^78–80^ Indeed, activation of toll-like receptor 9 with CpG oligodeoxynucleotides induced plasmacytoid DCs to secrete IFN-I, which stimulated NK cells to express cytolytic markers and increase tumour necrosis factor-related apoptosis-inducing ligand (TRAIL) and IFN-γ expression. This phenotypic switch significantly enhanced NK-cell mediated lysis of both GD2^+^ and GD2^–^ target neuroblastoma cells in vitro.^78^ NK cells represent a particularly attractive target in neuroblastoma due to their capacity for MHC-I independent target cell killing, using alternative interactions such as TRAIL to target tumour cells with cytolytic granules.^81^

### Cell-based NKT cell therapy

Cell-based therapies involving the adoptive transfer of chimeric-antigen receptor (CAR) T cells has revolutionized the treatment of haematological cancers, inducing striking and durable regressions of B-cell acute lymphoblastic leukaemia.^82 83^ Engineered CAR systems consist of antigen-binding domains fused to a T cell receptor (TCR)ϛς chain and additional co-stimulatory domains, such as those for CD28 or OX40.^82^ Multiple iterations of the technology have produced a potent method of targeting cancer cells for immune-cell mediated killing, independently of MHC function. However, the use of patient-derived autologous T cells is time consuming and susceptible to clinical manufacturing failures, making “off-the-shelf” allogeneic alternatives an attractive option.^84^ Vα24-invariant–natural killer T (iNKT) cells are an innate-like cell type which exhibit potent cytotoxicity and rapid cytokine secretion following the recognition of glycolipids presented by CD1d.^85^ Crucially, CD1d monomorphism overcomes human leukocyte antigen (HLA)-restricted graft-versus-host disease usually seen with allogeneic transfer of T cells, making iNKT cells a promising and readily available CAR-T system which may benefit wider use of the therapy.^84^ Preclinical studies have demonstrated the efficient trafficking of NKT cells to tumours such as neuroblastomas in response to tumour-derived chemokines.^86^ Furthermore, infiltration of iNKT cells into neuroblastomas evokes anti-tumour immunity by targeting and killing CD1d-expressing TAMs.^87^ Observations that intratumoural abundance of iNKT cells is a favourable prognostic indicator in neuroblastoma^86^ supported efforts to study the effect of adoptively transferred activated cells on disease progression.

A first-in-human phase I clinical trial (NCT03294954) administered chimeric antigen receptor NKT (CAR-NKT) cells to children with refractory neuroblastoma.^88^ After administration, the CAR-NKT persisted in vivo, trafficked to tumours and induced objective responses and metastasis regression in a subset of patients.^88^ Another phase I study (NCT00877110) tested a combination of the anti-GD2 antibody m3F8 and adoptive transfer of haploidentical NK cells for high-risk refractory neuroblastoma.^89^ The study demonstrated that combination treatment was well tolerated and induced anti-tumour responses in a subset of patients.

## Targeting the cyclic GMP-AMP synthase-stimulator of interferon genes pathway

**Figure 3:**
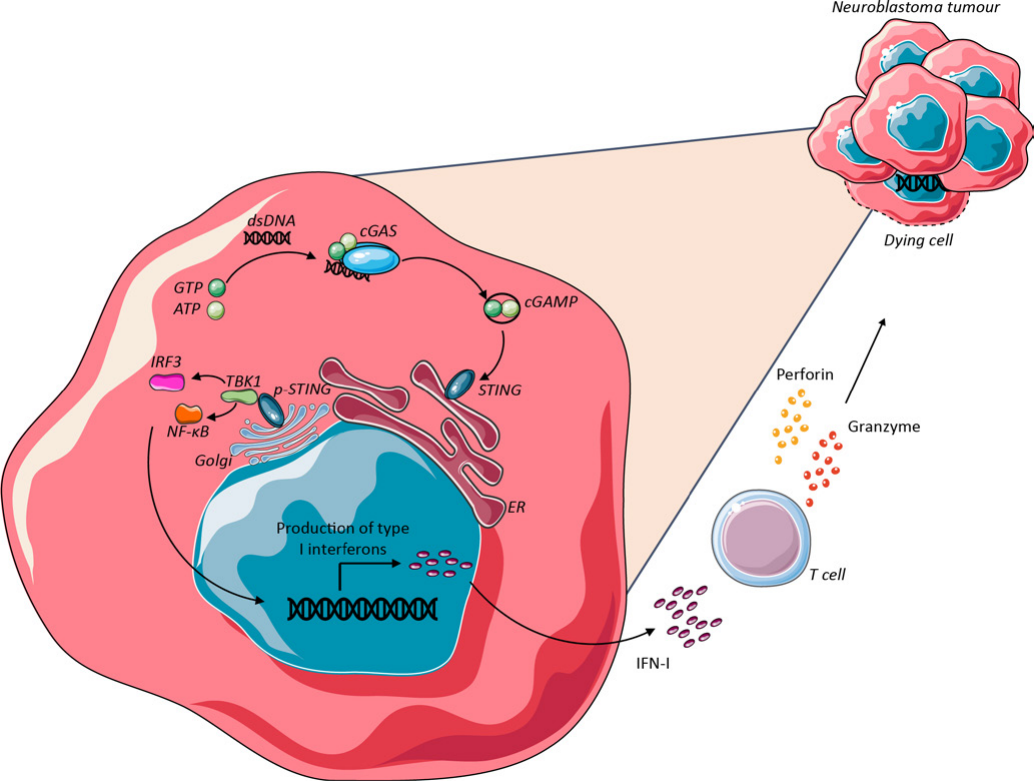
The STING pathway activation against neuroblastoma. Radiation therapy causes damage to cancer cells leading to cell death and release of double stranded DNA (dsDNA). Tumour cell-derived dsDNA binds to cyclic GMP-AMP synthase (cGAS) leading to the production of cyclic GMP-AMP (cGAMP) and subsequent activation of the stimulator of interferon genes (STING). Activated STING undergoes ER to Golgi translocation and activates tank binding kinase (TBK1), interferon regulatory factor (IRF3) and the transcription regulator NF-κB. These trigger pro-inflammatory gene expression, recruitment of CD8^+^ T cells and promote anti-tumour cytotoxic activity. (Adapted from Garland *et al*,^111^ Saulters *et al*^112^). dsDNA, double-stranded deoxyribonucleic acid; ER, endoplasmic reticulum; IFN, interferon; NF-κB, nuclear factor kappa-light-chain-enhancer of activated B cells; pSTING, phosphorylated stimulator of interferon genes .

The cyclic GMP-AMP synthase-stimulator of interferon genes (cGAS-STING) pathway is an intracellular DNA sensing pathway and integral innate immune defense mechanism to viral infection or cellular transformation. On detection of pathogen-associated molecular patterns, such as cytoplasmic DNA (cDNA), STING signaling drives IFN-I production which can boost anti-tumour effector T-cell function (figure 3).^90 91^

The effect of STING agonism on tumour-immune interactions has been studied in multiple cancer types, including pancreatic cancer, breast cancer, head and neck cancer, lung cancer and leukaemia.^91^ Multiple preclinical studies have demonstrated beneficial therapeutic responses to dimethylxanthone acetic acid (DMXAA) and cyclic dinucleutides (CDNs) in transgenic models of pancreatic ductal adenocarcinoma, in addition to promising combination strategies with gemcitabine and ICIs.^92 93^ Intraperitoneal administration of DMXAA induced IFN-I production by activation of the tank binding kinase/interferon regulatory factor pathway and multimodal therapy with radiation promoted inflammation in the TME, reducing T-cell tolerance of pancreatic tumours.^94^

STING activation and poly-ADP ribose polymerase (PARP) inhibition was investigated in breast cancer gene (BRCA)-deficient breast cancer models; the combination significantly improved anti-tumour responses by driving IFN-I production, T-cell recruitment and DC activation, while inducing immunological memory and reducing adaptive resistance to PARP inhibitors (PARPi).^95^ In addition to the effect on T cells, reprogramming of TAMs towards anti-tumour M1 phenotypes has also been observed following CDN treatment in lymphoma^96^ and breast cancer models.^97^ Interestingly, the PARPi and CDN combination was more efficacious than an alternative strategy of PARPi with macrophage depletion, suggesting macrophage repolarization can be more beneficial than outright deletion.^97^

Radiotherapy also induces STING activation through the accumulation of DNA damage and triggering of DNA damage responses.^98^ One preclinical study found that treatment of irradiated non-small cell lung cancer tumours with the VEGF inhibitor anlotinib led to impaired DNA damage response, accumulation of cDNA and activation of STING signaling.^99^ This enhanced CD8^+^ T-cell recruitment and anti-tumour effector responses, while synergising with ICB to inhibit tumour growth significantly more than irradiation alone.^99^ In summary, the cGAS-STING pathway is recognized as a potent innate immune mechanism responsible for detecting tumour-derived DNA and triggering immune responses against tumours. Synthetic STING agonists, used in conjunction with ICIs, cancer vaccines and adoptive T-cell transfer therapies are emerging as promising therapeutic options with the potential to enhance the effectiveness of cancer treatments and improve patient outcomes.

### STING agonists in neuroblastoma treatment

Around 50% of high-risk neuroblastomas exhibit *MYCN* amplification^100^ which is associated with reduced IFN-I pathway activity and insensitivity to ICB, therefore activating the STING pathway may be beneficial in targeting *MYCN* amplified neuroblastoma tumours. A study using genetically engineered mouse models of neuroblastoma used the STING agonist DMXAA to evaluate the anti-tumour effect of dual STING activation and *MYCN* inhibition.^101^ While *MYCN-*amplified tumours are associated with reduced IFN-I pathway activity in primary metastatic neuroblastomas and established cellular models, depletion of N-Myc potently augmented IFN pathway activity in response to DMXAA treatment.^101^ Another study evaluated cytosolic CDN administration to overcome the issues with membrane impermeability; after cytosolic administration CDNs activated STING and IFN-I production, and induced expression of anti-tumour cytokines and chemokines, causing tumour cell apoptosis.^102^ STING signaling in neuroblastoma is poorly characterized and not yet studied in clinical trials. In addition, due to their inherent instability, the majority of CDN-based STING agonists currently under clinical development are normally administered directly into the tumour site.^103^ CDNs also face obstacles such as limited intracellular bioavailability and suboptimal pharmacokinetic properties. These factors pose challenges that hinder their effectiveness and limit their clinical applicability to tumours that are easily accessible. To address this caveat, recent research endeavors have focused on generating STING-activating compounds that are amenable to systemic delivery or STING-activating nanoparticles designed to efficiently deliver the endogenous STING ligand into the cytosol.^102^ Such an approach has been explored in preclinical models of neuroblastoma that led to inhibition of tumour growth and improved response to ICB.^102^ However, the utility of STING activating compounds against neuroblastoma remains to be explored in clinical trials.

## Concluding remarks

Targeting neuroblastoma with immunotherapies has been challenging because of its highly immunosuppressive TME, with some promising treatments not translating successfully (see Outstanding questions). The use of checkpoint inhibitors as a treatment for neuroblastoma has not been successful in the clinic and any studies that have been conducted only proved the safety of using these inhibitors in children, suggesting that they might need to be administered in combination with other treatments. The most widely studied and successful immunotherapy is the use of anti-GD2 antibodies and approaches to overcome resistance to GD2 treatments and improve response are currently being explored. Combination of anti-GD2 and anti-CD47 antibodies showed promising results in mouse models and has now been evaluated in a first-in-human clinical trial.^68^ Moreover, the use of GD2 antibodies in combination with CAR-NKT cells was shown to induce tumour regression in a phase I clinical trial.^88^ Use of CAR-T cells for childhood solid tumours has not been effective, however, the use of dual CAR-T cells targeting both GD2 and B7-H3 is being explored.^104^ This approach has been successful in a mouse model, leading to anti-tumour response and avoidance of tumour cell escape because of heterogeneity in antigen abundance. It was also proven that these CAR-T cells did not reach an exhaustive state easily, had increased metabolic fitness and a favourable safety profile with decreased neurotoxicity.^104^ These recent approaches are directed towards developing effective treatment regimens that use innovative immunotherapies and reduce toxic traditional chemotherapies. Furthermore, exquisite expression of certain proteins in neuroblastoma owing to the unique developmental origin, such as GD2 or L1-CAM, may also be exploited as distinct and exclusive targets for next generation immunotherapies. Discovery and exploitation of novel targets may underpin efficacious neuroblastoma therapies in future. However, promising ongoing studies into known targets may soon reveal mechanisms and opportunities for rational combination strategies with currently available chemotherapy, radiotherapy, and immunotherapy.

## Outstanding questions

Many questions still remain for the use of immunotherapies in neuroblastoma treatment. Combination strategies involving anti-GD2 have been successful in clinical trials, but have not yet received approval for clinical use. Furthermore, the limited impact of ICIs in the clinic and strategies to improve patient responses need further investigation. The possibility of using radiotherapy in combination with STING agonists also needs to be explored. Moreover, possible combination treatments to enhance responses need to be tested and the safety and efficacy of dual CAR-T cells needs to be tested in a clinical setting in paediatric oncology.

## Resources

NCT03363373: https://www.clinicaltrials.gov/ct2/show/NCT03363373

NCT01757626: https://clinicaltrials.gov/ct2/show/NCT01757626

NCT00072358: https://clinicaltrials.gov/ct2/show/NCT00072358

NCT00492167: https://clinicaltrials.gov/ct2/show/NCT00492167

NCT02914405: https://clinicaltrials.gov/ct2/show/NCT02914405

NCT01704716: https://clinicaltrials.gov/ct2/show/NCT01704716

NCT04751383: https://clinicaltrials.gov/ct2/show/NCT04751383

NCT03189706: https://clinicaltrials.gov/ct2/show/NCT03189706

NCT04501757: https://clinicaltrials.gov/ct2/show/NCT04501757

NCT03033303: https://clinicaltrials.gov/ct2/show/NCT03033303

NCT02650648: https://clinicaltrials.gov/ct2/show/NCT02650648

NCT01662804: https://clinicaltrials.gov/ct2/show/NCT01662804

NCT01419834: https://clinicaltrials.gov/ct2/show/NCT01419834

NCT03332667: https://clinicaltrials.gov/ct2/show/NCT03332667

NCT01711554: https://clinicaltrials.gov/ct2/show/NCT01711554

NCT02573896: https://clinicaltrials.gov/ct2/show/NCT02573896

NCT03786783: https://clinicaltrials.gov/ct2/show/NCT03786783

NCT01857934: https://clinicaltrials.gov/ct2/show/NCT01857934

NCT04385277: https://clinicaltrials.gov/ct2/show/NCT04385277

NCT00026312: https://www.clinicaltrials.gov/ct2/show/NCT00026312

NCT01445379: https://clinicaltrials.gov/ct2/show/NCT01445379

NCT02813135: https://clinicaltrials.gov/ct2/show/NCT02813135

NCT02304458: https://clinicaltrials.gov/ct2/show/NCT02304458

NCT02914405: https://clinicaltrials.gov/ct2/show/NCT02914405

NCT04412408: https://clinicaltrials.gov/ct2/show/NCT04412408

NCT04730349: https://clinicaltrials.gov/ct2/show/NCT04730349

NCT05302921: https://clinicaltrials.gov/ct2/show/NCT05302921

NCT02541604: https://clinicaltrials.gov/ct2/show/NCT02541604

NCT00877110: https://clinicaltrials.gov/ct2/show/NCT00877110

NCT03209869: https://clinicaltrials.gov/ct2/show/NCT03209869

NCT03294954: https://clinicaltrials.gov/ct2/show/NCT03294954

NCT03242603: https://clinicaltrials.gov/ct2/show/NCT03242603
